# Evolutionary History of the *Clostridium difficile* Pathogenicity Locus

**DOI:** 10.1093/gbe/evt204

**Published:** 2013-12-11

**Authors:** Kate E. Dingle, Briony Elliott, Esther Robinson, David Griffiths, David W. Eyre, Nicole Stoesser, Alison Vaughan, Tanya Golubchik, Warren N. Fawley, Mark H. Wilcox, Timothy E. Peto, A. Sarah Walker, Thomas V. Riley, Derrick W. Crook, Xavier Didelot

**Affiliations:** ^1^Nuffield Department of Clinical Medicine, Oxford University, John Radcliffe Hospital, United Kingdom; ^2^National Institute for Health Research, Oxford Biomedical Research Centre, John Radcliffe Hospital, Oxford, United Kingdom; ^3^Microbiology and Immunology, School of Pathology and Laboratory Medicine, The University of Western Australia, Crawley, WA, Australia; ^4^Division of Microbiology and Infectious Diseases, PathWest Laboratory Medicine, Nedlands, WA, Australia; ^5^Department of Microbiology, John Radcliffe Hospital, Oxford, United Kingdom; ^6^Department of Statistics, University of Oxford, United Kingdom; ^7^Department of Microbiology, The General Infirmary, Old Medical School, Leeds, United Kingdom; ^8^Leeds Institute of Molecular Medicine, University of Leeds, United Kingdom; ^9^Department of Infectious Disease Epidemiology, Imperial College, Norfolk Place, London, United Kingdom

**Keywords:** *Clostridium difficile*, pathogenicity locus, PaLoc, bacterial evolution, toxin, mobile genetic element

## Abstract

The symptoms of *Clostridium difficile* infection are caused by toxins expressed from its 19 kb pathogenicity locus (PaLoc). Stable integration of the PaLoc is suggested by its single chromosomal location and the clade specificity of its different genetic variants. However, the PaLoc is variably present, even among closely related strains, and thus resembles a mobile genetic element. Our aim was to explain these apparently conflicting observations by reconstructing the evolutionary history of the PaLoc. Phylogenetic analyses and annotation of the regions spanning the PaLoc were performed using *C. difficile* population-representative genomes chosen from a collection of 1,693 toxigenic (PaLoc present) and nontoxigenic (PaLoc absent) isolates. Comparison of the core genome and PaLoc phylogenies demonstrated an eventful evolutionary history, with distinct PaLoc variants acquired clade specifically after divergence. In particular, our data suggest a relatively recent PaLoc acquisition in clade 4. Exchanges and losses of the PaLoc DNA have also occurred, via long homologous recombination events involving flanking chromosomal sequences. The most recent loss event occurred ∼30 years ago within a clade 1 genotype. The genetic organization of the clade 3 PaLoc was unique in containing a stably integrated novel transposon (designated Tn6218), variants of which were found at multiple chromosomal locations. Tn6218 elements were Tn916-related but nonconjugative and occasionally contained genes conferring resistance to clinically relevant antibiotics. The evolutionary histories of two contrasting but clinically important genetic elements were thus characterized: the PaLoc, mobilized rarely via homologous recombination, and Tn6218, mobilized frequently through transposition.

## Introduction

Mobile genetic elements represent a diverse group of evolutionarily successful parasitic DNA sequences, capable of transfer across phylogenetic distances well beyond the usual scope of homologous recombination (Forsberg et al. 2012). The abundance of certain elements among bacteria reflects an ability to catalyze their own spread and to confer a selective advantage on their host by transmitting beneficial accessory genes (Roberts and Mullany 2009; Wellington et al. 2013). These may encode toxins or resistance to antimicrobials that may result in severe clinical phenotypes. Well characterized examples include the CTXphi bacteriophage carrying the cholera toxin genes in *Vibrio cholerae* (Chun et al. 2009), the integrons propagating metallo-β-lactamase genes among Gram-negative bacteria (Cornaglia et al. 2011), and the SCCmec element conferring resistance to methicillin in *Staphylococcus aureus* (Enright et al. 2002).

The impact of mobile DNA on pathogen evolution is further illustrated by hypervirulent strains of the Gram-positive anaerobe *Clostridium difficile*, which are both toxigenic and multidrug resistant (McDonald et al. 2005; Tenover et al. 2012). *Clostridium difficile* is the cause of a significant world-wide nosocomial and community disease burden, particularly affecting the elderly (Miller et al. 2010; Bauer et al. 2011). The disease manifestations of *C. difficile* infection range from mild diarrhea to toxic megacolon and death (Karas et al. 2010). Disease results from the effects of two large clostridial toxins designated A and B, encoded by the genes *tcdA* and *tcdB* (Kuehne et al. 2010). These genes are contained in a 19-kb pathogenicity locus (PaLoc) (Braun et al. 1996), which is present in the genomes of toxigenic strains and absent from their nontoxigenic, nondisease-causing counterparts. The PaLoc contains three further genes: *tcdR* encoding a RNA polymerase sigma factor that positively regulates toxin expression (Mani and Dupuy 2001), *tcdC* considered (now controversially) a corresponding negative regulator (Hundsberger et al. 1997; Matamouros et al. 2007; Cartman et al. 2012; Bakker et al. 2012), and *tcdE*, which is related to bacteriophage holins (Tan et al. 2001). This relationship suggests that the PaLoc may derive at least some sequences from temperate bacteriophages, a hypothesis supported by the influence of some *C. difficile* phages on toxin gene expression (Govind et al. 2009).

The population structure of *C. difficile* consists of five clades, each of which includes toxigenic strains (Griffiths et al. 2010; Dingle et al. 2011; Stabler et al. 2012). When present, the PaLoc is always found at the same chromosomal location (Braun et al. 1996; Dingle et al. 2011), and because it lacks a recombinase gene, it is tempting to conclude that the PaLoc was stably integrated before the clades diverged. However, nontoxigenic strains are present throughout the *C. difficile* population, occasionally sharing the same multilocus sequence type (ST) as toxigenic strains (Dingle et al. 2011). This irregular distribution resembles that of a mobile genetic element. Phylogenetic reconstructions based on short fragments of the *tcdB* and *tcdC* genes have shown that toxigenic strains from the same clade tend to have a similar PaLoc, but that the inter-clade PaLoc relationships differ from typical chromosomal genes (Dingle et al. 2011). Intriguingly, 115 bp and 7.2 kb sequences of unknown origin have been observed at the PaLoc chromosomal insertion site in nontoxigenic strains (Braun et al. 1996; Elliott et al. 2009). These sequences are absent from toxigenic strains. This study aimed to reconstruct the evolutionary history of the PaLoc and to understand the significance of a novel recombinase-containing PaLoc insertion.

## Materials and Methods

### Ethics Statement

This study included bacterial isolates for which no corresponding patient data were used. The isolates were collected without written informed consent as part of studies of *C. difficile* transmission for which permission was obtained from Berkshire Ethics Committee (10/H0505/83), the UK National Information Governance Board (8-05(e)/2010), and Oxfordshire Research Ethics Committee (ref:09/H0606/80) (infant isolates). There is no requirement under Australian law to seek consent for the use of anonymized bacterial isolates for research.

### Isolates and Genome Sequencing

Isolates were cultured from *C. difficile*-positive stool samples identified by enzyme immunoassay (Premier Toxins A&B Enzyme Immunoassay; Meridian Bioscience Europe, Naples, Italy) at the Clinical Microbiology Laboratory, Oxford University Hospitals NHS Trust, Oxford, or by cytotoxin testing at the Leeds Teaching Hospitals NHS Trust, Leeds.

Isolates Q6 and Q24, and ES248, were sent to Perth from diagnostic laboratories in Queensland and Victoria, respectively, for molecular typing. All other Australian isolates were from Western Australia and recovered by toxigenic culture of stool samples, apart from WA12 which was recovered from a positive blood culture (Elliott et al. 2009). Isolates were referred to as toxigenic if a phenotypic toxin detection test on the stool from which they were cultured was positive and the PaLoc was present in the genome. Isolates were referred to as nontoxigenic if their genome lacked the PaLoc. Nontoxigenic isolates could be isolated from a toxin positive stool if a mixed infection involving a PaLoc-positive isolate was present.

Oxford isolates (*n* = 1,224) were first cultured between September 16, 2006, and March 3, 2012, Leeds isolates (*n* = 365) between December 20, 2005, and March 12, 2008, and Australian isolates (*n* = 34) between May 14, 1980, and October 31, 2010. An additional 21 Oxford clinical isolates cultured from ELISA-negative stool samples between January 19, 2011, and September 7, 2011, were included, together with 39 isolates from healthy and symptomatic Oxford infants isolates cultured between November 1, 2008, and January 6, 2012 (Stoesser et al. 2011), 8 PCR-ribotype reference isolates, strain 8864 (Soehn et al. 1998), and a Canadian ST122 isolate Opt2249 isolated in 2009, representing a putative sixth genetic lineage (Knetsch et al. 2012). The overall total was 1,695 isolates (including two genomes available in GenBank FN668375 and FN665652; He et al. 2010). Culture and genotyping by multilocus sequence typing (MLST) and PCR-ribotyping were performed as described (Griffiths et al. 2010). The notation ST1(027) was adopted to indicate Sequence Type 1 (PCR-ribotype 027). PCR-ribotypes corresponding to STs are indicated in supplementary table S1, Supplementary Material online (where known), and listed in our previous study (Dingle et al. 2011). Genomes were sequenced as described previously (Didelot et al. 2012; Dingle et al. 2012) using Illumina sequencing by synthesis technology (Bentley et al. 2008) and Velvet de novo assemblies were made (Zerbino and Birney 2008). VelvetOptimiser 2.1.7 (with Velvet 1.0.7–1.0.18) was run to find the optimal Kmer size (*k*) for each sample and the N50 (length of the smallest contig such that all contigs of that length or less form half of the final assembly), as well as the expected coverage (average kmer coverage of contigs) and coverage cutoff (kmer coverage threshold) (supplementary tables S1 and S4, Supplementary Material online). These genomes provided a large denominator from which a smaller number of population-representative genomes were chosen for detailed study of the PaLoc and nontoxigenic strains, thus ensuring that the known *C. difficile* population was properly represented and avoiding unnecessary duplication. This overall depth of sampling also facilitated the detailed study of specific genotypes which were of interest (supplementary table S4, Supplementary Material online) due to the occurrence of relevant recent evolutionary events.

### Gene Prediction, Annotation, and Comparison

Putative open reading frames within the PaLoc variants, PaLoc insertion sites, and mobile genetic elements studied in detail (figs. 5–7 and supplementary figs. S4 and S6, Supplementary Material online) were identified using Artemis genome browser and annotation tool (Rutherford et al. 2000). BlastN, BlastP, and TBlastN searches of putative genes and predicted translation products against GenBank (http://www.ncbi.nlm.nih.gov/blast/Blast.cgi?, last accessed December 20, 2013) using the default settings were used to predict possible functions using the relationship of the sequences to known genes and proteins (supplementary tables S2 and S3, Supplementary Material online). Bacterial Isolate Genome Sequence Database (BIGSdb) (Jolley and Maiden 2010) was used to perform BlastN and BlastP searches of the 1,695 genomes using loci identified using the above approach. Sequence comparisons were performed using Artemis Comparison Tool (Carver et al. 2005) and alignments using Clustal Omega (Sievers et al. 2011). DNA secondary structure predictions were generated using the RNAfold web server at http://rna.tbi.univie.ac.at/cgi-bin/RNAfold.cgi (last accessed December 20, 2013) (Gruber et al. 2008).

### Phylogenetic Analyses

**Fig. 1.—:**
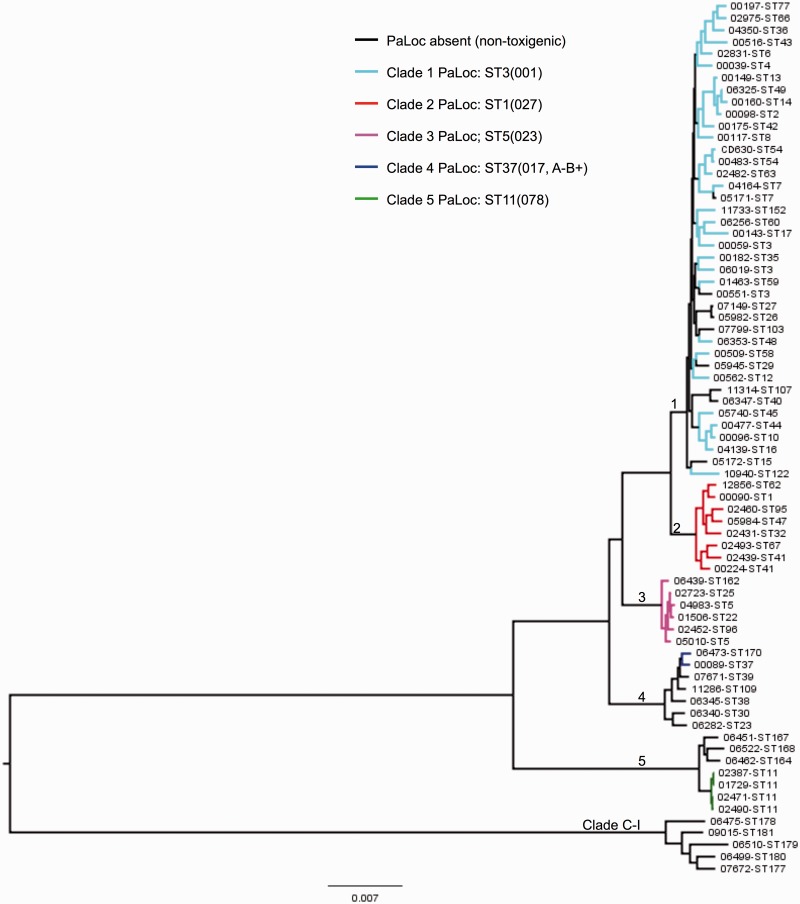
Phylogenetic relationship between toxigenic and nontoxigenic *Clostridium difficile* isolates. Maximum likelihood tree generated from the genomes of 72 representative isolates, plus CD630 (Sebaihia et al. 2006). Clades are indicated by their designated number. Nontoxigenic isolates are indicated by black branches. Toxigenic isolates are indicated by branches colored according to clade. The ST and PCR-ribotype (in brackets) of a well characterized representative of each clade is indicated.

A total of 73 isolates (including reference CD630, Sebaihia et al. 2006) were chosen for global phylogenetic analysis (fig. 1 and supplementary table S1, Supplementary Material online); they represented the *C. difficile* population structure and included both toxigenic and nontoxigenic strains. The congruence of population structure as defined by MLST (Dingle et al. 2011) and by the core genome (fig. 1) provides validation for this approach to isolate choice. At least one representative of each available ST belonging to clades 2, 3, 4, and 5 was included, together with a subset of the more numerous but relatively genetically homogeneous (Dingle et al. 2011) clade 1 STs (*n* = 40). This approach ensured that the figures derived from analysis of these isolates were clear, rather than overly dominated by clade 1. The 73 isolates represented extremes of clinical severity (Walker et al. 2013), abundance, and nontoxigenic strains (Dingle et al. 2011). More than one genome of the same ST was analyzed when the genotype existed in both toxigenic and nontoxigenic forms (ST7 and ST3) or the ST could be further discriminated on the basis of PCR-ribotype (ST3, ST5, ST11, and ST41) in which case one example of each ST-ribotype combination was included (fig 1 and supplementary table S1, Supplementary Material online). When more than one genome was available for a given ST, the genome used was chosen on the basis of its assembly quality particularly in the region of the PaLoc (supplementary table S1, Supplementary Material online). Genomes representing additional toxigenic clade 1 STs did not further inform the study due to their very close genetic relationship with other members of this clade, and their exclusion facilitated ease of data interpretation. The overall relative abundance of each ST is not shown because this study was not an epidemiological survey (i.e., all isolates collected during the study period in each location were not included). Such data for the Oxford region have been reported previously for 1,290 isolates (Dingle et al. 2011).

**Fig. 2.—:**
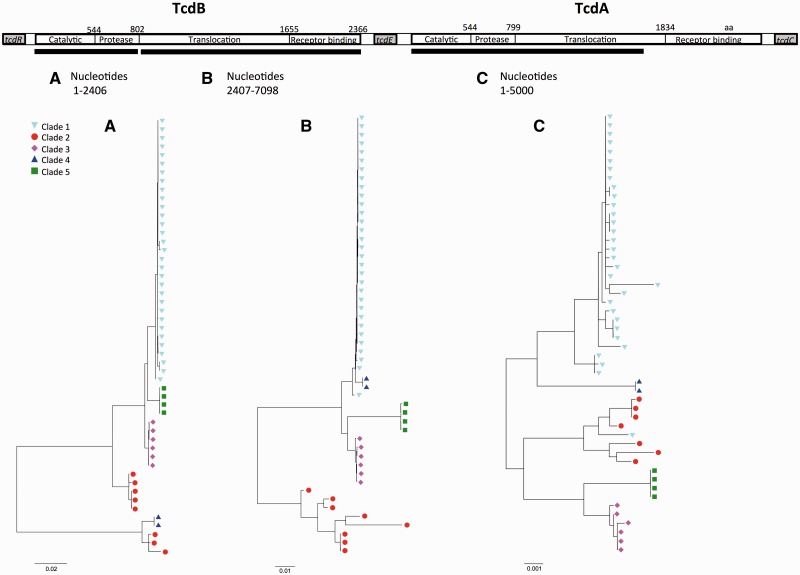
Cross-population phylogeny of the PaLoc. Phylogenies constructed from the catalytic and protease domains of *tcdB* (*A*), from the translocation and receptor binding domains of *tcdB* (*B*), and from the catalytic, protease, and part of the translocation domain of *tcdA* (*C*). Breaks in assembly caused by repetitive sequences in the receptor-binding domain of *tcdA* precluded its inclusion. Colored shapes indicate clade as in figure 1. Strain labels and bootstrap values are shown in supplementary figure S2, Supplementary Material online.

The 73 genomes were used to construct a maximum likelihood tree using phyml (Guindon et al. 2010) (fig. 1), based on the raw concatenation of gene-by-gene Muscle alignments of 1,426 “core” genes (concatenated length of 1.2 Mbp), defined as the genes from the annotation of reference CD630 (Sebaihia et al. 2006) for which a homologous sequence was found in all 72 genomes using BlastN covering a minimum of 90% of the query sequence and with an *E*-value threshold of 10^−^^10^. Under these stringent conditions, we found only one such match in any genome for any queried gene, so we were confident that our procedure identified homologs rather than paralogs. Phylogenetic analysis of the PaLoc alone (fig. 2) was performed using MEGA version 5 (available from http://www.megasoftware.net/, last accessed December 20, 2013) to construct maximum likelihood trees (Tamura et al. 2011).

**Fig. 3.—:**
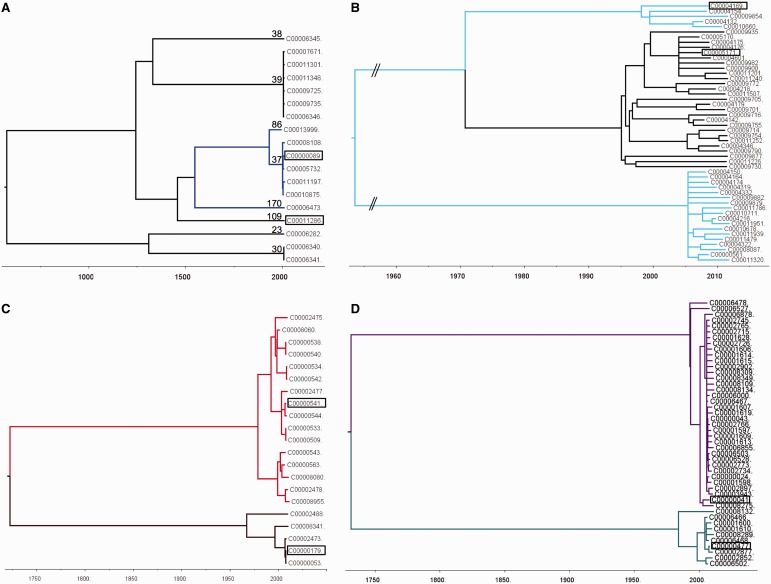
Dating PaLoc acquisition, loss, and exchange. (*A*) Time-scaled ClonalFrame tree dating the acquisition of the PaLoc by clade 4 to between 466 and 554 years ago. Genomes of 7 toxigenic isolates (blue) representing 3 STs (indicated above branches), including 2 from GenBank FN668375 (ST37, C00010875) and FN665652 (ST86, C00013999) (He et al. 2010), and 11 nontoxigenic isolates (black) representing 5 STs were included. (*B*) Time-scaled ClonalFrame tree dating the loss of the PaLoc in ST7 to between 1971 and 1995; 27 nontoxigenic genomes (black) and 23 toxigenic genomes (pale blue) were included. (*C*) Time-scaled ClonalFrame tree dating the exchange of clade 1 PaLocs within ST58 to between 208 and 417 years ago. Five genomes containing one PaLoc variant (green) and 16 containing the other (red) were included. (*D*) Time-scaled ClonalFrame tree dating the exchange of clade 1 PaLocs with ST44 to between 196 and 401 years ago; 36 genomes containing one PaLoc variant (purple) and 10 containing the other (turquoise) were included. The four pairs of genomes compared in figure 4 are boxed in each of parts (*A*)–(*D*).

**Fig. 4.—:**
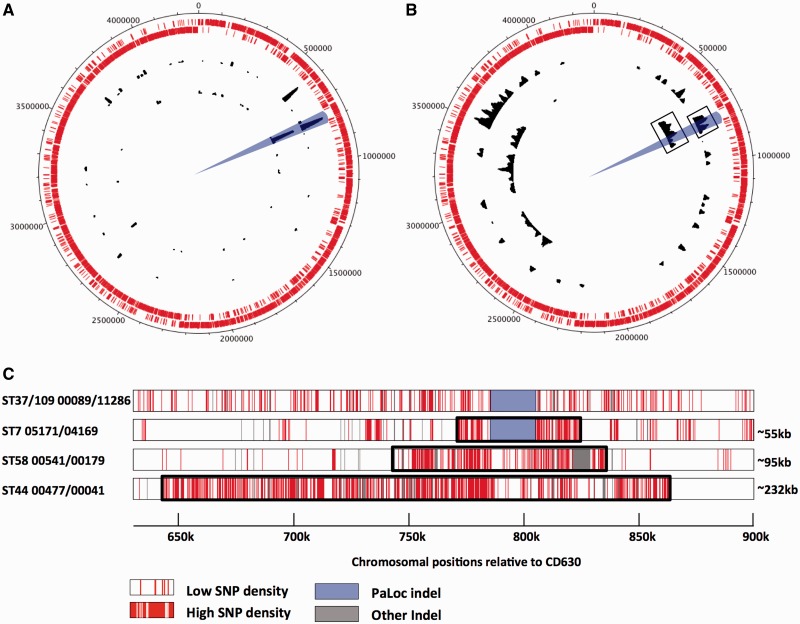
PaLoc acquisition, loss, and exchange by homologous recombination involving long fragments of chromosomal DNA. (*A*) PaLoc acquisition and loss. Whole-genome distributions of indels between the two pairs of isolates marked by boxes in figure 3*A* (toxigenic ST37 and nontoxigenic ST109 outer black ring) and 3*B* (toxigenic and nontoxigenic ST7 inner black ring). The location of the PaLoc is indicated by blue shading. The two outer rings composed of small red lines indicate the open reading frames annotated on the forward and reverse strands of reference genome CD630 (Sebaihia et al. 2006). (*B*) PaLoc exchange within clade 1. Whole-genome distributions of polymorphism between the two pairs of isolates marked by boxes in figure 3*C* (toxigenic ST58, outer black ring) and 3*D* (toxigenic ST44, inner black ring). (*C*) Distribution of polymorphism between the four pairs of genomes shown in (*A*) and (*B*) within the region of the genome containing the PaLoc. Each row represents a pairwise comparison, and polymorphisms are shown in red. Enlarging the region of the genome flanking the PaLoc in this way allowed the distribution of polymorphisms to be used to estimate the size of the recombination events (black boxes) as ∼55 kb replaced by ∼36 kb during PaLoc loss by ST7, and ∼95 kb or ∼232 kb during PaLoc exchange within ST58 and ST44.

Fine-scale phylogenies were constructed by comparing the alignments of the core genes of multiple genomes of clade 4 (fig. 3*A*) or multiple genomes sharing the same ST, namely, ST7(026), ST58(056), and ST44(015) (fig. 3*B*–*D*) and ST6(005), ST3(001), and ST54(012) (fig. 8). These phylogenies were constructed using ClonalFrame (Didelot and Falush 2007), with ancestry times measured in years by combining the known isolation dates of the genomes and a previous estimate of the *C. difficile* molecular clock based on pairwise comparisons of genomes longitudinally isolated from the same patients (Didelot et al. 2012). The polymorphisms between four pairs of isolates (highlighted in fig. 3) were mapped along the whole CD630 genome (Sebaihia et al. 2006) by aligning their de novo assemblies against CD630 using MuMMER version 3.23 (Kurtz et al. 2004). The distributions of these polymorphisms were then shown on a circular map of the whole CD630 genome using DNAplotter (Carver et al. 2009) (fig. 4*A* and *B*) and on a linear map ranging from position 630 k to 900 k of CD630 (fig. 4*C*). The same method was used to represent the distribution of the average pairwise distance between clade 1 isolates (supplementary fig. S3*A*, Supplementary Material online).

The maximum likelihood method ace implemented in the R package ape (Paradis et al. 2004) was used to jointly estimate the rate of gain or loss of the PaLoc and the ancestral state of each internal node of the genome-wide phylogeny (supplementary fig. S1, Supplementary Material online). As no statistical support was found for a more complex model, this reconstruction assumed a unique rate for both gain and loss throughout the phylogenetic tree shown in figure 1.

### Nucleotide Sequence Accession Numbers

**Fig. 5.—:**
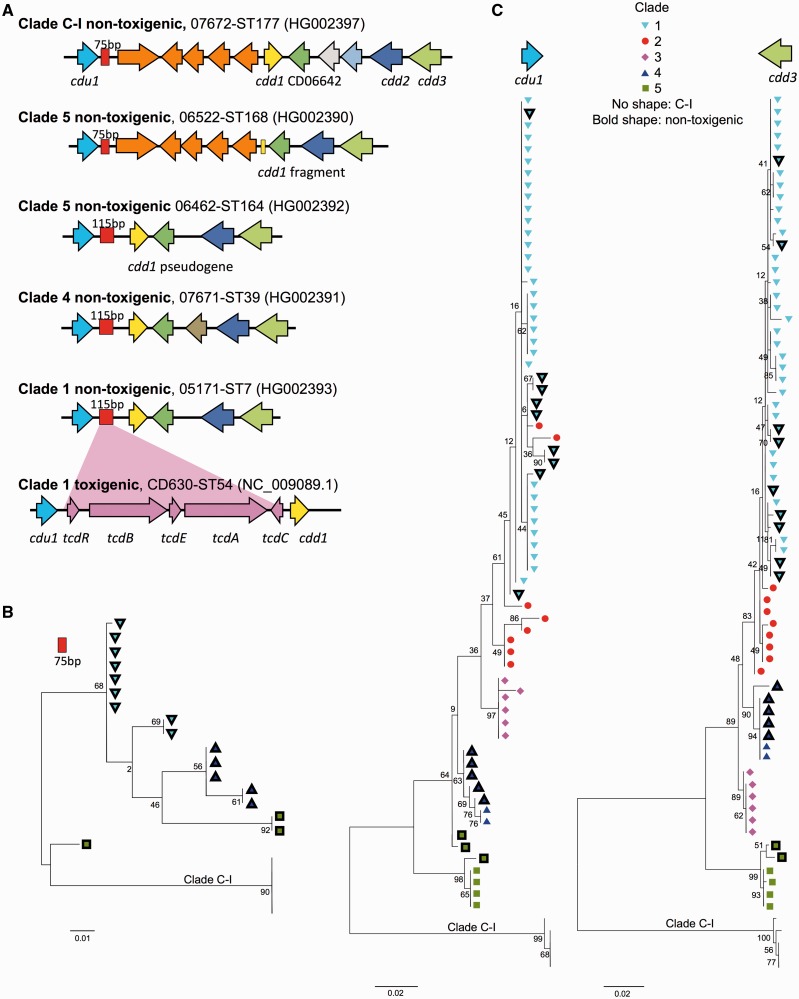
The chromosome flanking the PaLoc insertion site in nontoxigenic isolates follows the five clades population structure. (*A*) Schematic depiction of the PaLoc insertion site of nontoxigenic isolates representing the four clades in which they have been identified; from the top, clade C-I ST177, clade 5 ST168, clade 5 ST167, clade 4 ST39, and clade 1 ST7. The PaLoc (pink), which replaces 115 bp (red box) in toxigenic strains, is represented for ST7. The five genes identified in this location in a single clade 5 strain, WA12 (Elliott et al. 2009), are also found in clade C-I (orange). (*B*) Maximum likelihood tree constructed from the 75 bp of the “PaLoc replacing” 115 bp sequence common to all nontoxigenic isolates and indicated as a red box in (*A*). Bootstrap values are indicated. (*C*) Maximum likelihood trees constructed from the PaLoc flanking genes *cdu1* and *cdd3*, using the isolates shown in figure 1. These genes were chosen because they contained sufficient polymorphism to discriminate clades 1 and 2.

**Fig. 6.—:**
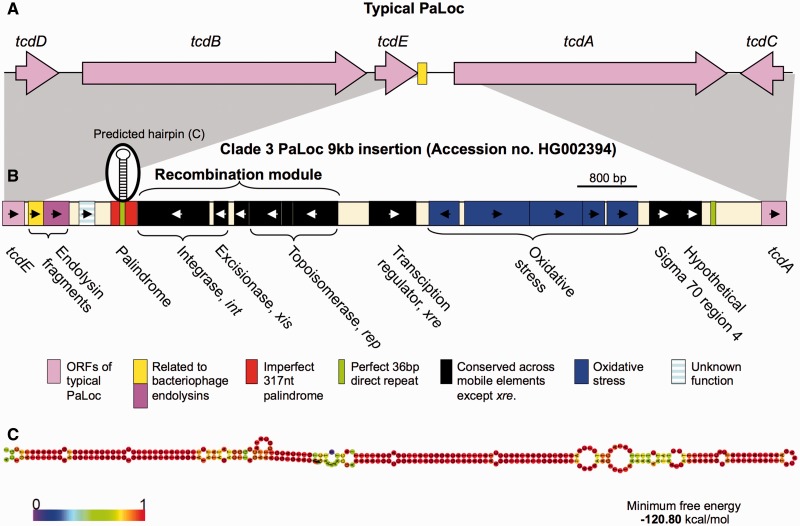
Genetic organization of the 9-kb insertion within the clade 3 PaLoc. (*A*) Schematic depiction of the genetic organization of a typical PaLoc as found in the reference genome CD630 (Sebaihia et al. 2006). A short fragment of endolysin-like sequence is indicated in yellow. (*B*) Genetic organization of the 9-kb clade 3 PaLoc insertion. Putative functions of the predicted genes were identified on the basis of Blast searches of GenBank. The orientation of each gene is indicated by an arrow. The endolysin, *int*, and *rep* genes were fragmented, hence they contain multiple arrows. The endolysin sequence found in the insertion is indicated in dark pink to distinguish it from the fragment common to typical PaLoc variants (yellow). The *int*, *xis*, and *rep* are referred to in the text as a recombination module. *xre* indicates a putative DNA-binding protein belonging to the xenobiotic (stress) response element family of transcriptional regulators. It occurs upstream of a gene cluster predicted to function in resisting oxidative stress. The genes showing homology to sigma 70 region 4 may be concerned with redirecting promoter recognition by the host RNA polymerase. The 3′ terminal gene is hypothetical but conserved among certain conjugative transposons. (*C*) Predicted hairpin structure formed by the 317 nt imperfect palindrome, generated using the RNAfold web server (http://rna.tbi.univie.ac.at/cgi-bin/RNAfold.cgi, last accessed December 20, 2013) (Gruber et al. 2008). The structure is oriented sideways, the top of hairpin to the right. The colors of the bases (as per the key) indicate their probability of being paired or unpaired as shown.

**Fig. 7.—:**
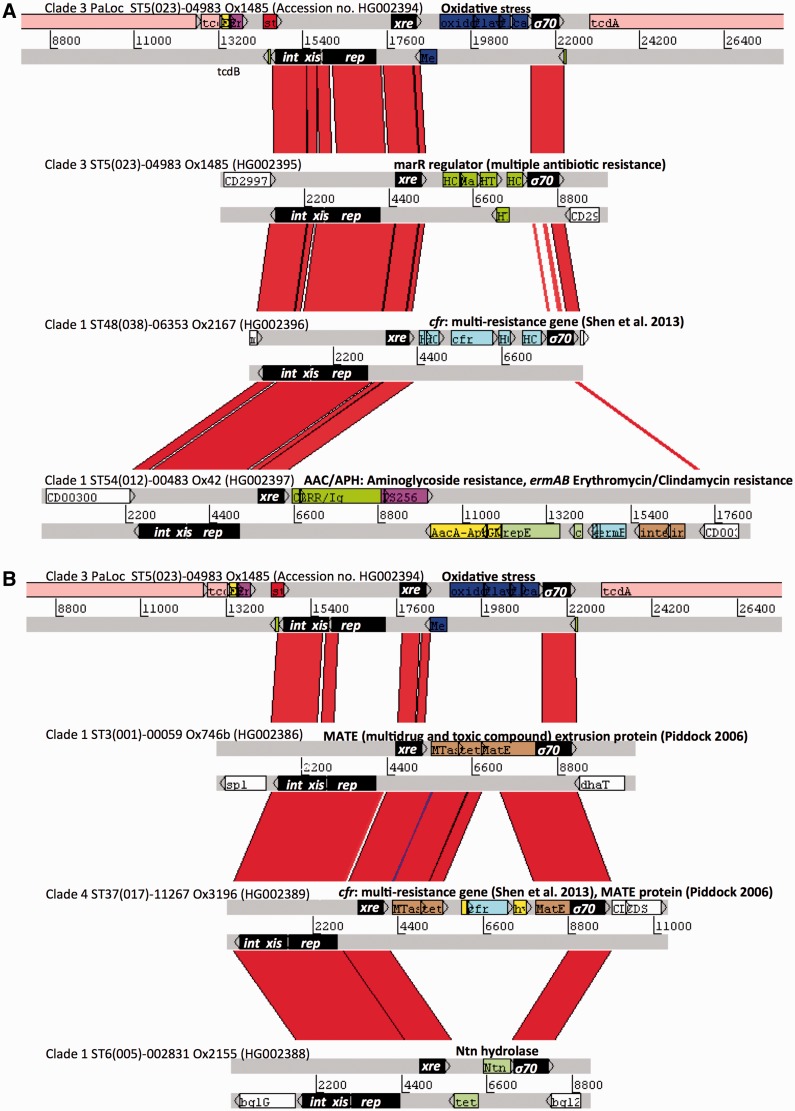
A group of closely related novel mobile genetic elements: Tn6218. (*A*) Comparison of three mobile elements with the clade 3 PaLoc (top, colored as in fig. 6) generated using the Artemis Comparison Tool (Carver et al. 2005). Genes shown in black are common to the mobile elements, PaLoc insertion (fig. 6), and conjugative transposons (supplementary fig. S4, Supplementary Material online). The accessory genes (colored) putatively confer resistance to polyketide antibiotics, chloramphenicol (*cfr*), aminoglycosides (*AacA-AphD*), and erythromycin (*ermAB*). (*B*) Comparison of three mobile elements with the clade 3 PaLoc (top), but the elements are distinguished from the PaLoc insertion by a distinct Rep protein variant. Accessory genes include a multidrug and toxic compound extrusion (MATE) family protein, *cfr*, and an N-terminal nucleophile (Ntn) hydrolase superfamily protein (includes penicillin acylase). BlastP data used to assign putative functions to accessory genes are summarized in supplementary table S2, Supplementary Material online. Accession numbers of the sequences submitted to European Nucleotide Archive are indicated.

**Fig. 8.—:**
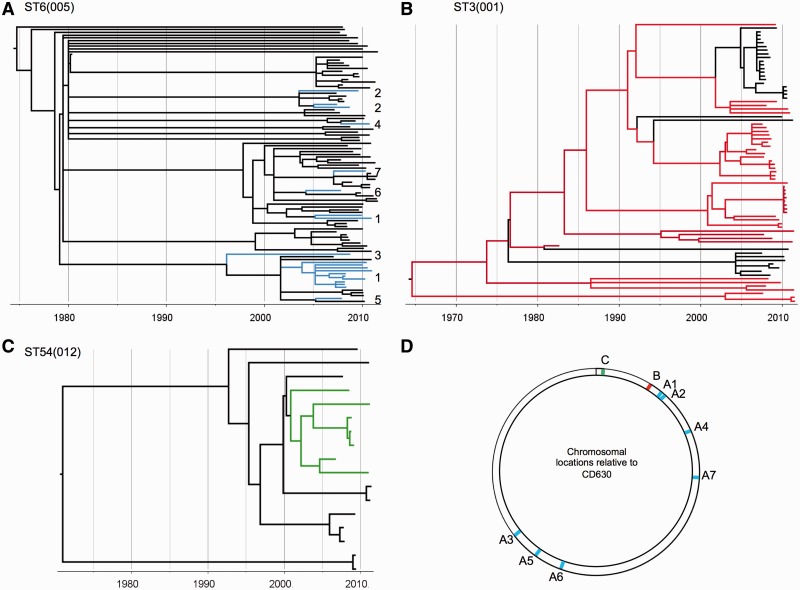
Evidence of recent transposition by three Tn6218 elements. Time-scaled ClonalFrame trees constructed using multiple genomes of the same ST. (*A*) Presence of the ST6(005) element (fig. 7*B*) is indicated by blue branches. The polymorphism of the element and its seven different chromosomal locations (*D*) are consistent with multiple independent insertion events occurring since 1995. (*B*) Presence of ST3(001) element (fig. 7*B*) is indicated by red branches; sequence identity of the element and its single chromosomal insertion site were consistent with one integration prior to 1970 and five subsequent losses. (*C*) Presence of ST54(012) element (fig. 7*A*) is indicated by green branches, consistent with a single insertion event around 2001. (*D*) Chromosomal locations of the elements shown in (*A*), (*B*), and (*C*) are colored accordingly.

The genomes which underwent detailed analysis have been submitted to the European Bioinformatics Institute short read archive. The project, accession numbers, and isolate details are listed in supplementary table S1, Supplementary Material online, for figures 1, 2, 4, 5, and 7, and in supplementary table S4, Supplementary Material online, for figures 3 and 8. The genomes can be obtained at http://www.ebi.ac.uk/ena/data/search? (last accessed December 20, 2013) Loci and mobile elements (together with the chromosomal junction sequences of the latter) which underwent detailed annotation (figs. 5–7) were submitted to the European Nucleotide Archive and accession numbers are indicated in the figures. They can be accessed at http://www.ebi.ac.uk/ena/data/view/accessionnumber (last accessed December 20, 2013).

## Results

### Isolates and Genomes

The whole-genome sequences of 1,619 toxigenic *C. difficile* isolates were examined: 1,207 and 364 from UK clinical cases in Oxford and Leeds, respectively, 15 from asymptomatic or symptomatic Oxfordshire infants (Stoesser et al. 2011), 26 from infections occurring in Australia, 3 PCR-ribotype reference isolates, strain 8864 (Soehn et al. 1998), an example of ST122 previously described as clade 6 (Knetsch et al. 2012), and 2 clade 4 genomes from GenBank (FN665652 and FN668375) (He et al. 2010).

The genomes of 76 nontoxigenic isolates were also studied: 38 from Oxford patients, 1 from a patient in Leeds, 24 from asymptomatic or symptomatic Oxfordshire infants (Stoesser et al. 2011), 8 from Australian human infections (including WA12, Elliott et al. 2009), and 5 PCR-ribotype reference isolates. All isolates were genotyped by MLST, the ST being determined either by conventional sequencing (Griffiths et al. 2010) using the database available at http://pubmlst.org/Cdifficile (last accessed December 20, 2013) or extracted bioinformatically from the genomes. ST designations were consistent by both methods. A total of 83 unique STs were represented among the toxigenic genomes and 22 among the nontoxigenic genomes. Consistent with previous findings (Dingle et al. 2011), all genomes of an individual ST were either toxigenic or nontoxigenic with the exception of ST7, ST3, and ST48.

### Eventful Evolutionary History of the PaLoc

The distribution of the PaLoc within the population was assessed by building a phylogenetic tree based on whole-genome sequences of a representative of each of the 22 nontoxigenic STs plus 51/83 toxigenic STs spanning all previously described *C. difficile* clades (Dingle et al. 2011; Knetsch et al. 2012; Stabler et al. 2012) (fig. 1 and supplementary table S1, Supplementary Material online). This tree featured a novel, highly divergent lineage containing only nontoxigenic strains, which was designated C-I. Clade C-I contained five STs represented by four Australian isolates and one from Oxford, UK. The remaining 17 nontoxigenic STs occurred alongside toxigenic variants in clades 1, 4, and 5 (fig. 1). The overall distribution of nontoxigenic genomes suggested that the ancestral *C. difficile* population may have lacked the PaLoc, and that multiple independent PaLoc acquisitions and losses have since occurred. To investigate this, a maximum likelihood ancestral state reconstruction was performed using the genomes of the same 73 isolates (Paradis et al. 2004). This estimated a high rate of PaLoc acquisition or loss (159 per unit of branch length with standard error 41), corresponding to an expected total of 26 events throughout the tree. With such a high evolutionary rate, it was not possible to infer whether the ancestor of the five toxigenic clades was toxigenic or not because several gain and loss events would have occurred since (supplementary fig. S1, Supplementary Material online).

The global PaLoc evolutionary history was investigated further by reconstructing its phylogeny using the 51 toxigenic genomes included in figure 1 (fig. 2 and supplementary fig. S2, Supplementary Material online). Neighbor-joining trees for the functional domains of *tcdA* and *tcdB* (Davies et al. 2011) showed that although members of the same clade almost always clustered closely together, the relationship between the clades was different to the core genome (fig. 1). Furthermore, these relationships changed across the PaLoc for clades 2 and 4, consistent with previous observations (Sambol et al. 2000). The phylogeny of the PaLoc itself was therefore consistent with multiple, clade-specific acquisitions after each clade had already undergone some degree of clonal expansion. Such post clade-divergence acquisition was further supported by the decrease in average pairwise distances across the clade 1 PaLoc relative to its flanking chromosomal sequences (supplementary fig. S3*A*, Supplementary Material online); if the clade 1 PaLoc was acquired after the lineage had diverged, one would expect its PaLoc to exhibit this lower level of polymorphism. It is possible that the ancestral *C. difficile* was nontoxigenic, that the nontoxigenic strains in clades C-I, 4, and 5 have never acquired the PaLoc, whereas at least some of the nontoxigenic strains in clade 1 may have lost the PaLoc at some point after its acquisition.

### Specific Instances of PaLoc Acquisition, Loss, and Exchange

A relatively recent acquisition of the PaLoc by previously nontoxigenic strains was suggested by the PaLoc distribution in clade 4 (fig. 1). To confirm this, a whole-genome time-scaled ClonalFrame tree (Didelot and Falush 2007) was constructed which dated the PaLoc acquisition to approximately 500 years ago, between 1459 and 1547 (fig. 3*A*). However, this may be an underestimate because a short-term molecular clock was used to date a relatively ancient event. The short-term molecular clock estimate we used (Didelot et al. 2012) is in good agreement with another short-term estimate in *C. difficile* (He et al. 2013), but two orders of magnitude higher than a previous long-term estimate (He et al. 2010). Similar dependency of the clock rate, on the evolutionary timescale at which it is measured, has been described in other organisms and can be theoretically explained, for example, by purifying selection slowly purging mutations that are slightly deleterious (Ho et al. 2007; Morelli et al. 2010; Didelot et al. 2012).

A more ancient acquisition of the clade 1 PaLoc (fig. 2) was indicated by its clade-wide distribution and the possibility of several subsequent losses (fig. 1). Phylogenetic comparison of clade 1 ST7 toxigenic (*n* = 23) and nontoxigenic (*n* = 27) genomes using a time-scaled ClonalFrame tree (Didelot and Falush 2007; Didelot et al. 2012) confirmed that a single PaLoc loss took place ∼30 years ago between 1971 and 1995 (fig. 3*B*). Two recent PaLoc exchanges were also identified within clade 1 ST44 and ST58, which were dated to approximately 290 and 280 years ago (fig. 3*C* and *D*).

These recent gain, loss, and exchange events were visualized at the chromosomal scale by plotting the distribution of indels (fig. 4*A*) and SNPs (fig. 4*B*) among four pairs of isolates, one pair taken from each tree in figure 3. By zooming in to the region containing the PaLoc, SNP plots indicated that for the three most recent events, very long chromosomal fragments of ∼55, ∼95, and ∼232 kb have been exchanged (fig. 4*C*). This suggests that host-mediated homologous recombination is the mechanism underlying recent PaLoc loss and exchange. Such long recombination events appear to be clade-specific, because the phylogenies of genes flanking the PaLoc (fig. 5*A* and *C*) and 75 bp of the 115 bp PaLoc-replacing sequence common to all nontoxigenic strains (fig. 5*A* and *B*) (Braun et al. 1996) were congruent with the genome-wide phylogeny. Interestingly, the two lower rows of figure 4*C* contain fewer SNPs than the flanking sequences, indicating that further PaLoc exchange may have occurred after the large recombination event, or the region of the chromosome containing the PaLoc evolves at a slower rate. Further PaLoc losses and exchanges were less clearly discernable among the large number of genomes studied due to the impact of subsequent evolutionary events such as SNP accumulation and shorter recombinations. The length of PaLoc acquisition events resembled the PaLoc itself, as indicated by the lower polymorphism of the clade 1 PaLoc relative to flanking sequences (supplementary fig. S3*A*, Supplementary Material online) and the lack of a recombination signature extending beyond the PaLoc in clade 4 (top row in fig. 4*C*). Imperfect direct repeats were found flanking the PaLoc when cross-population comparisons of the insertion sites were performed (supplementary fig. S3*B*, Supplementary Material online).

### Atypical Genetic Organization of the Clade 3 PaLoc

Population-wide comparisons of the PaLoc genetic organization revealed a previously undocumented 9-kb insertion in clade 3 (*n* = 83 isolates) located between *tcdE* and *tcdA* (fig. 6*A* and *B*). The five STs in clade 3 spanned significant phylogenetic (fig. 1) and geographic distances (UK and Australia), indicating that the insertion was neither recent nor geographically localized. A recombination module was identified within the insertion, which comprised a tyrosine recombinase (*int*, catalyses integration and excision), its cognate excisionase (*xis*), and a putative topoisomerase (*rep*, replication initiation factor). Results of BlastP searches against GenBank to assign these putative functions are summarized in supplementary table S2, Supplementary Material online. An imperfect inverted repeat of length 317 nt was located adjacent to the recombination module. This palindrome was predicted to form an energetically stable hairpin (fig. 6*C*). Fragments of putative endolysin-related sequences (supplementary table S2, Supplementary Material online) spanned the 5′ terminal junction of the insertion with the typical PaLoc. A putative transcription regulatory gene similar to regulators involved in the xenobiotic response (*xre*) occurred upstream of genes, which were predicted to confer resistance to oxidative stress (supplementary table S2, Supplementary Material online).

### A Novel Group of Mobile Genetic Elements

The presence of stop codons in the *int* and *rep* genes of the clade 3 PaLoc insertion indicated a probable loss of function (fig. 6*B*). However, BlastN searches of the recombination module (*int-xis-rep*) against the whole genomes identified many closely related mobile elements (>90% nucleotide sequence identity) in which these reading frames were intact (fig. 7). The elements were homologous to parts of the clade 3 PaLoc insertion located between two perfect 36-bp direct repeats (green boxes in fig. 6*B*), one of which occurred in the middle of the palindrome at the top of the hairpin structure. These elements have been assigned the designation Tn6218 using the transposon registry located at http://www.ucl.ac.uk/eastman/research/departments/microbial-diseases/tn (last accessed December 20, 2013). Although the genomes were unclosed, the 5′ and 3′ junctions of intact Tn6218 elements contiguous with flanking chromosomal DNA were identified successfully and annotated by comparison with reference genome CD630 (Sebaihia et al. 2006) (fig. 7 and http://www.ebi.ac.uk/ena/data/view/accessionnumber, last accessed December 20, 2013). The accession numbers of the Tn6218 elements are listed in figure 7, adjacent to their corresponding element.

Four genes (*int*, *xis*, *rep*, and *xre*) were common to all Tn6218 elements (fig. 7*A* and *B*) although some had a distinct *rep* variant, which was only distantly related at the amino acid level and lacked the N-terminal *xre*-like sequence (fig. 7*B*). The *int*, *xis*, and *rep* genes (and two variably present 3′ terminal ORFs, fig. 7) shared 37–84% amino acid identity with conjugative transposon Tn916 (Flannagan et al. 1994; Roberts and Mullany 2009) (supplementary fig. S4 and table S3, Supplementary Material online). A variety of accessory genes conferring resistance to stress were present, including antimicrobials (fig. 7 and supplementary table S2, Supplementary Material online). Exposure to antibiotics, including fluoroquinolones and clindamycin, is likely an important risk factor either for the selection of *C. difficile* strains and/or the induction of *C. difficile* infections (Johnson et al. 1999; Loo et al. 2005; Kelly 2012; Deshpande et al. 2013). Some Tn6218 accessory genes are therefore likely to be clinically relevant; for example, the *ermB* gene (fig. 7*A* and supplementary table S2, Supplementary Material online) confers high-level resistance to clindamycin (Spigaglia et al. 2011), and a MATE family protein (multidrug and toxic compound extrusion, fig. 7*B* and supplementary table S2, Supplementary Material online) is predicted to confer resistance to fluoroquinolones and other drugs (Piddock 2006). The multidrug resistance gene *cfr* which confers resistance to several antimicrobial classes (Shen et al. 2013) was identified in both Tn6218 variants (fig. 7*A* and *B* and supplementary table S2, Supplementary Material online).

In common with Tn916 (Roberts and Mullany 2009), the Tn6218 chromosomal insertion sites were AT rich, and their 5′ and 3′ termini exhibited the direct and inverted repeats characteristic of integrated transposons (supplementary fig. S5*A*, Supplementary Material online). Evidence of repeated recent (<30 years) chromosomal gain and loss of three elements was obtained using time-scaled ClonalFrame trees (Didelot and Falush 2007; Didelot et al. 2012) constructed from large numbers of same ST genomes (fig. 8).

## Discussion

Phylogenetic analysis of whole-genome sequences representing the known *C. difficile* population structure revealed that the PaLoc has a complex evolutionary history. Each lineage acquired its current PaLoc variant after divergence, as indicated by the distinctive PaLoc phylogeny (fig. 2 and supplementary fig. S2, Supplementary Material online) and its low polymorphism relative to the flanking chromosome in clade 1 (supplementary fig. S3*A*, Supplementary Material online). Consistent with this, we identified a novel highly divergent lineage which appeared entirely nontoxigenic. It is arguable whether this clade represents a new species, subspecies, or simply an independent lineage, hence it was designated C-I in analogy with the cryptic clades of *Escherichia coli* (Luo et al. 2011). Our data suggest that the common ancestor of all modern *C. difficile* may have been nontoxigenic, although we cannot rule out an alternative scenario where ancient PaLoc integration was followed by losses and clade-specific PaLoc recombination events. The evolutionary events causing PaLoc gain, loss, and exchange preclude accurate reconstruction of the ancestral state (figs. 3 and 4 and supplementary fig. S1, Supplementary Material online).

The presence of a non-PaLoc insertion at its genomic location throughout divergent clade C-I and in one clade 5 recombinant (Elliott et al. 2009) (fig. 5*A*) reflects the high plasticity of the *C. difficile* genome (Sebaihia et al. 2006; He et al. 2010), which is also evident in the stable 9-kb insertion within the clade 3 PaLoc (fig. 6). Our data confirm previously reported (Sambol et al. 2000; Stabler et al. 2008) mosaicism within *tcdB* (fig. 2 and supplementary fig. S2, Supplementary Material online) and indicate that in addition to the primary PaLoc acquisition(s), exchanges and losses have occurred (figs. 1, 3, and 4).

The availability of whole genomes revealed the likely mechanism underlying recent PaLoc exchange and loss in clade 1 to be intra-clade homologous recombination involving sequences up to 232 kb (figs. 3 and 4). Such long fragments of chromosomal DNA could be introduced following transfer by integrated mobile elements, as proposed to explain similarly large (≤147 kb) recombination events within clade 2 ST1(027) (He et al. 2013). There is currently no evidence that the PaLoc region recombines any differently, in terms of mechanism or frequency, to other chromosomal loci (fig. 4*B* and *C*, He et al. 2013). In contrast, PaLoc acquisition by nontoxigenic strains seems to have involved DNA sequences close in size to the PaLoc itself, indicated by the congruence of the PaLoc flanking genes with core rather than the PaLoc phylogeny (fig. 5*C*) and the dip in polymorphism across the clade 1 PaLoc (supplementary fig. S3*A*, Supplementary Material online). Also, the relatively recent clade 4 PaLoc acquisition did not yield an obvious signature of recombination extending beyond the PaLoc (fig. 3*A* and 4*C*). For these reasons, site-specific recombination catalyzed by an integrase supplied *in trans* could be the mechanism of initial PaLoc acquisition. The absence of a perfect PaLoc integration site in clade C-I could explain its nontoxigenic status (supplementary fig. S3*B*, Supplementary Material online), but only five genomes of this clade are available to date, and toxigenic clade C-I strains may be discovered in future. The imperfect direct repeats that flank the PaLoc are consistent with site-specific recombination as a mechanism of PaLoc acquisition (supplementary figure S3*B*, Supplementary Material online). However, the replacement of 115 bp in nontoxigenic strains on primary PaLoc integration (Braun et al. 1996) requires a 115 bp excision to occur during this process (supplementary fig. S3*C*, Supplementary Material online). This could indicate that PaLocs are acquired by homologous recombination involving very short flanking sequences, but this hypothesis seems less likely given the precision and equivalence of each PaLoc insertion event (supplementary fig. S3*B*, Supplementary Material online), assuming multiple independent acquisitions.

It is likely that the nontoxigenic *C. difficile* population remains incompletely characterized, because toxigenic isolates, as dictated by laboratory detection methods and clinical importance, represent the vast majority of strains cultured to date. Consequently, the recent UK human toxigenic *C. difficile* population is likely to have been sufficiently sampled in the present study for reliable conclusions to be drawn. The number of toxigenic genotypes identified per clade varies widely (fig. 1) (Dingle et al. 2011; Knetsch et al. 2012; Stabler et al. 2012), consistent with the hypothesis of several independent PaLoc acquisitions followed by subsequent clonal expansions perhaps reflecting the time elapsed since acquisition. Clade 1, with the greatest diversity of toxigenic genotypes, may exemplify the most ancient acquisition and clades 4 and 5 the most recent, as indicated by their limited genotypic diversity (fig. 1). A relatively ancient PaLoc acquisition by clade 1 would also explain the emergence of nontoxigenic strains within this clade, as sufficient time has elapsed for occasional PaLoc losses to occur (figs. 3*B* and 4*A*). This provides an alternative explanation to the suggestion that the multiplicity of toxigenic genotypes detected in clade 1 reflects an unfortunate choice of MLST loci (Knetsch et al. 2013) (also refuted by the agreement of core phylogenies defined by MLST [Dingle et al. 2011] and whole genomes, fig. 1). An alternative explanation for the predominance of toxigenic clade 1 genotypes could be superior adaptation to the clinical environment, such as innate or acquired resistance to antibiotics.

The presence of toxin genes related to *tcdA* and *tcdB* in other *Clostridium* species including *C. sordellii* and *C. novyi* (Green et al. 1995; Just and Gerhard 2004) indicates the potential for PaLoc gene acquisition and diversification by inter-species recombination. This may explain the divergent regions of *C. difficile tcdB* (fig. 2, Sambol et al. 2000), which could impact on clade-associated clinical phenotypes (Walker et al. 2013) particularly in clade 4. Here, the altered substrate specificity of the divergent TcdB catalytic domain (fig. 2 and supplementary fig. S2, Supplementary Material online; Chaves-Olarte et al. 1999; Sambol et al. 2000) offers a possible explanation for the distinctive effect of clade 4 on clinical biomarkers (Walker et al. 2013). The identification of nontoxigenic *C. sordellii* variants (Walk et al. 2011) provides a further parallel with *C. difficile* and indicates that their evolutionary histories could potentially interconnect.

Previously described PaLoc variants of clades 1, 2, 4, and 5 share a common genetic organization, with the exception of occasional deletions in *tcdA* and *tcdC*, and a 1.1-kb insertion in clade 2 strain 8864 (Hammond and Johnson 1995; Soehn et al. 1998; Kato et al. 1999; Sebaihia et al. 2006). Characterization of the entire clade 3 PaLoc revealed a novel clade-wide 9-kb insertion (fig. 6), which may contribute to the relatively mild clinical phenotype of this clade (Walker et al. 2013). The central 8 kb of the insertion corresponded to a novel transposable element designated Tn6218. Identification of multiple Tn6218 sequences as PaLoc-independent mobile elements occupying a wide variety of chromosomal locations (figs. 7 and 8) excludes the possibility that this sequence represents an ancestral bacteriophage-like PaLoc sequence (additional to *tcdE*; Tan et al. 2001) and suggests its acquisition (together with the flanking 1.2 kb) soon after the PaLoc was acquired by clade 3. Tn6218 variants were widespread among the *C. difficile* population and carried multiple accessory genes (fig. 7), suggesting their frequent exchange or acquisition. Some of these genes are known to confer high-level resistance to clinically relevant antimicrobials, for example, *ermB* and clindamycin (Spigaglia et al. 2011; Kelly 2012; Deshpande et al. 2013) (supplementary table S2, Supplementary Material online). The multidrug resistance gene *cfr* is present in a wide range of Gram-positive and Gram-negative species (Shen et al. 2013), but it was identified in *C. difficile* for the first time here, in both Tn6218 variants (fig. 7*A* and *B*). The *cfr* gene could explain the resistance of certain *C. difficile* isolates to clindamycin in the absence of *ermB* (Spigaglia et al. 2011).

A mechanism whereby the Tn6218 elements could be excised from relatives of the clade 3 PaLoc insertion became apparent when the PaLocs of clade 3 and the clade 2 strain 8864 were compared (supplementary fig. S5*B*, Supplementary Material online). The central 8 kb of the clade 3 insertion was flanked by perfect 36-bp repeats and contained half the hairpin. The smaller 1.1-kb PaLoc insertion of strain 8864 (Soehn et al. 1998) lacked the Tn6218-like sequence but contained a single copy of the 36-bp repeat and the other half of the hairpin. This suggests a possible mechanism whereby these mobile elements could have evolved by excision of large unstable palindromes via recombination between two such direct repeats. This model is supported by data describing palindrome excision in *E. coli* (Leach 1994), and it would explain the origin of the 5′ and 3′ terminal inverted repeats of the Tn6218-like element (supplementary fig. S5*A* and *B*, Supplementary Material online).

To our knowledge, Tn6218 elements are the only transposons identified to date having a Tn916-like tyrosine recombinase but lacking the machinery of conjugation. Like Tn916 (Roberts and Mullany 2009), Tn6218 requires AT-rich insertion sites (supplementary fig. S5*A*, Supplementary Material online). Evidence of recent transposition was obtained (fig. 8), including that of the *ermB* ST54(012) element (fig. 8*C*), but it is unclear whether this occurs independently of conjugative transposons, which may mobilize Tn6218 elements *in trans* (Adams et al. 2002). Searches of GenBank identified Tn6218-like elements in the unannotated genomes of *Clostridium* sp. HGF2 (supplementary fig. S6*A*, Supplementary Material online), *Bifidobacteriumbreve* (supplementary fig. S6*B*, Supplementary Material online), *Ruminococcus*, *Lachnospiraceae*, and *Coprobacillus* sp. indicating the likelihood of interspecies transposition.

The population distribution of the PaLoc contrasts with that of Tn6218 (fig. 8) and indicates that this essential virulence determinant is not easily transmitted among *C. difficile* isolates in the manner of a typical mobile genetic element. However, the continually evolving relationship between the PaLoc and *C. difficile* is apparent; nontoxigenic strains provide a reservoir of potential toxigenic strains, and recent PaLoc acquisitions and exchanges indicate the need for awareness that new toxigenic strains may emerge in the future.

## Supplementary Material

Supplementary figures S1–S6 and tables S1–S4 are available at *Genome Biology and Evolution* online (http://www.gbe.oxfordjournals.org/).

Supplementary Data
