# Supplementary material for: Evolutionary History of the Clostridium difficile Pathogenicity Locus
Source: Genome Biol Evol. 2013 Dec 11;6(1):36–52. doi: 10.1093/gbe/evt204 (PMC3914685; doi:10.1093/gbe/evt204)
Supplement: Supplementary Data [file supp_6_1_36__index.html]

Evolutionary History of the Clostridium difficile Pathogenicity Locus — Evolutionary History of the Clostridium difficile Pathogenicity Locus — Supplementary Data 

# Evolutionary History of the *Clostridium difficile* Pathogenicity Locus

## Supplementary Data

files

**Files in this Data Supplement:**

- Supplementary Data - tiff file
- Supplementary Data - tiff file
- Supplementary Data - tiff file
- Supplementary Data - tiff file
- Supplementary Data - tiff file
- Supplementary Data - tiff file
- Supplementary Data - xlsx file
- Supplementary Data - xls file
- Supplementary Data - xlsx file
- Supplementary Data - xlsx file
