# Supplementary figures and images for: Evolutionary History of the Clostridium difficile Pathogenicity Locus
Source: Genome Biol Evol. 2013 Dec 11;6(1):36–52. doi: 10.1093/gbe/evt204 (PMC3914685; doi:10.1093/gbe/evt204)

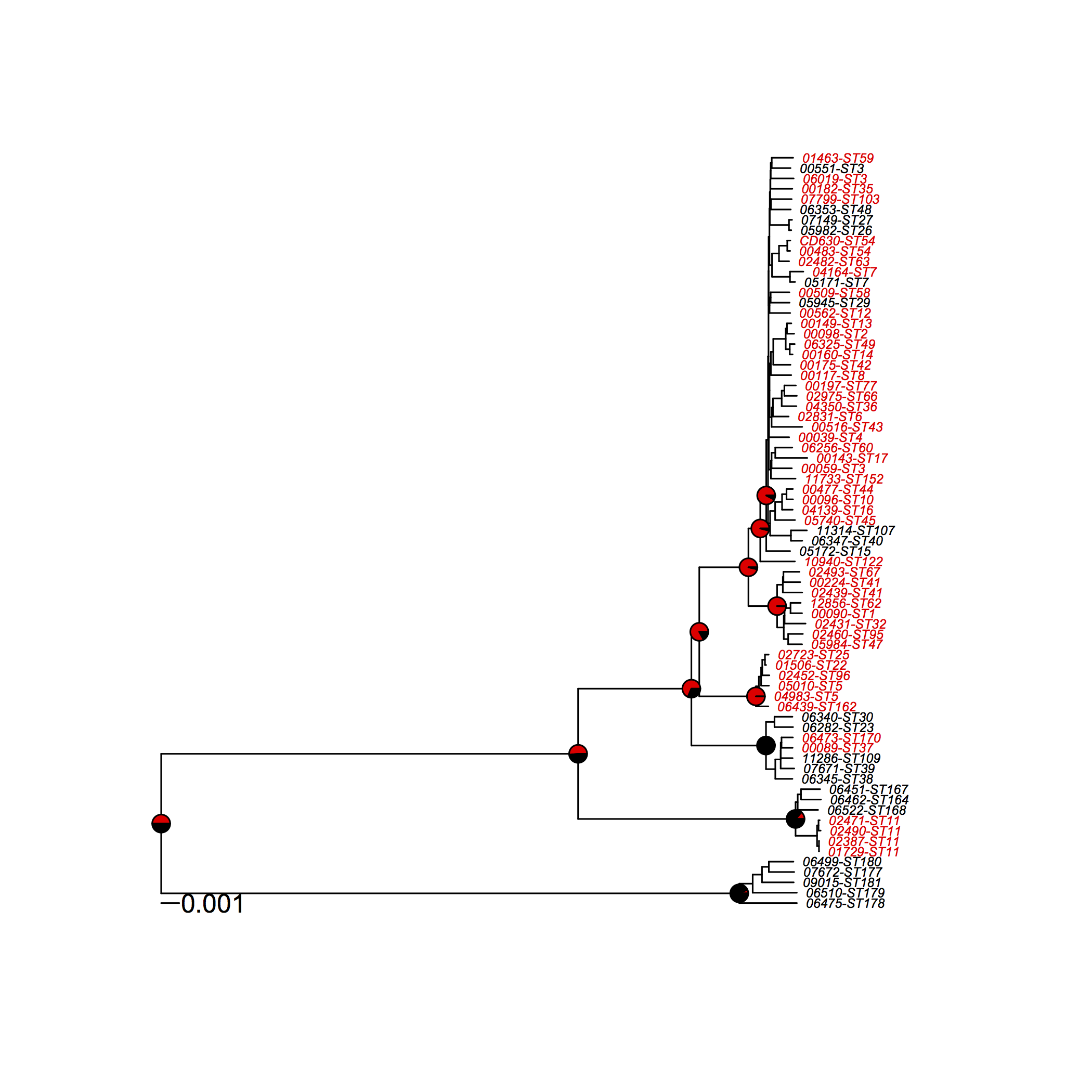

Supplement: Supplementary Data [file supp_evt204_Figure_S1_final.tif]

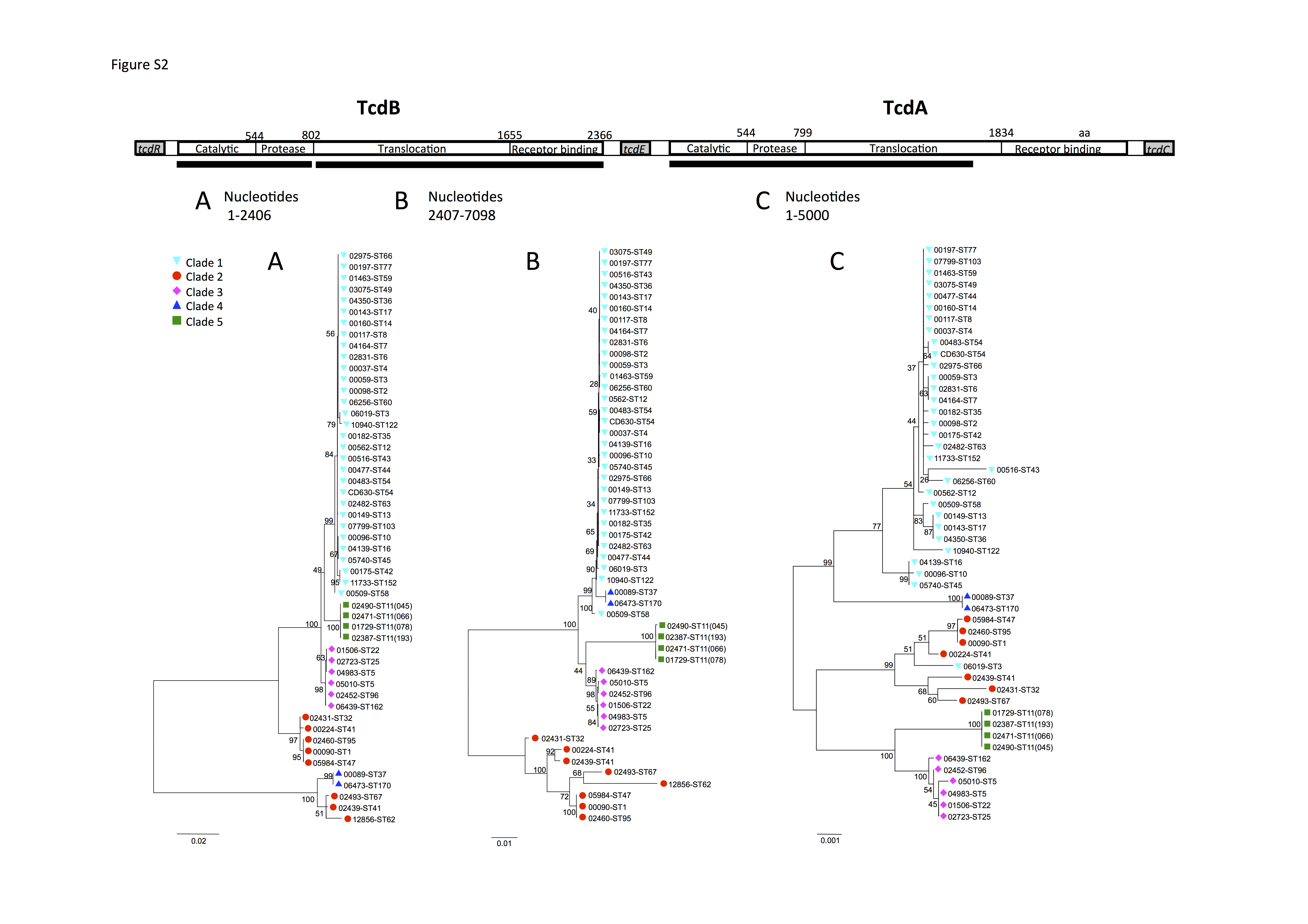

Supplement: Supplementary Data [file supp_evt204_Figure_S2_revised.ppt.tif]

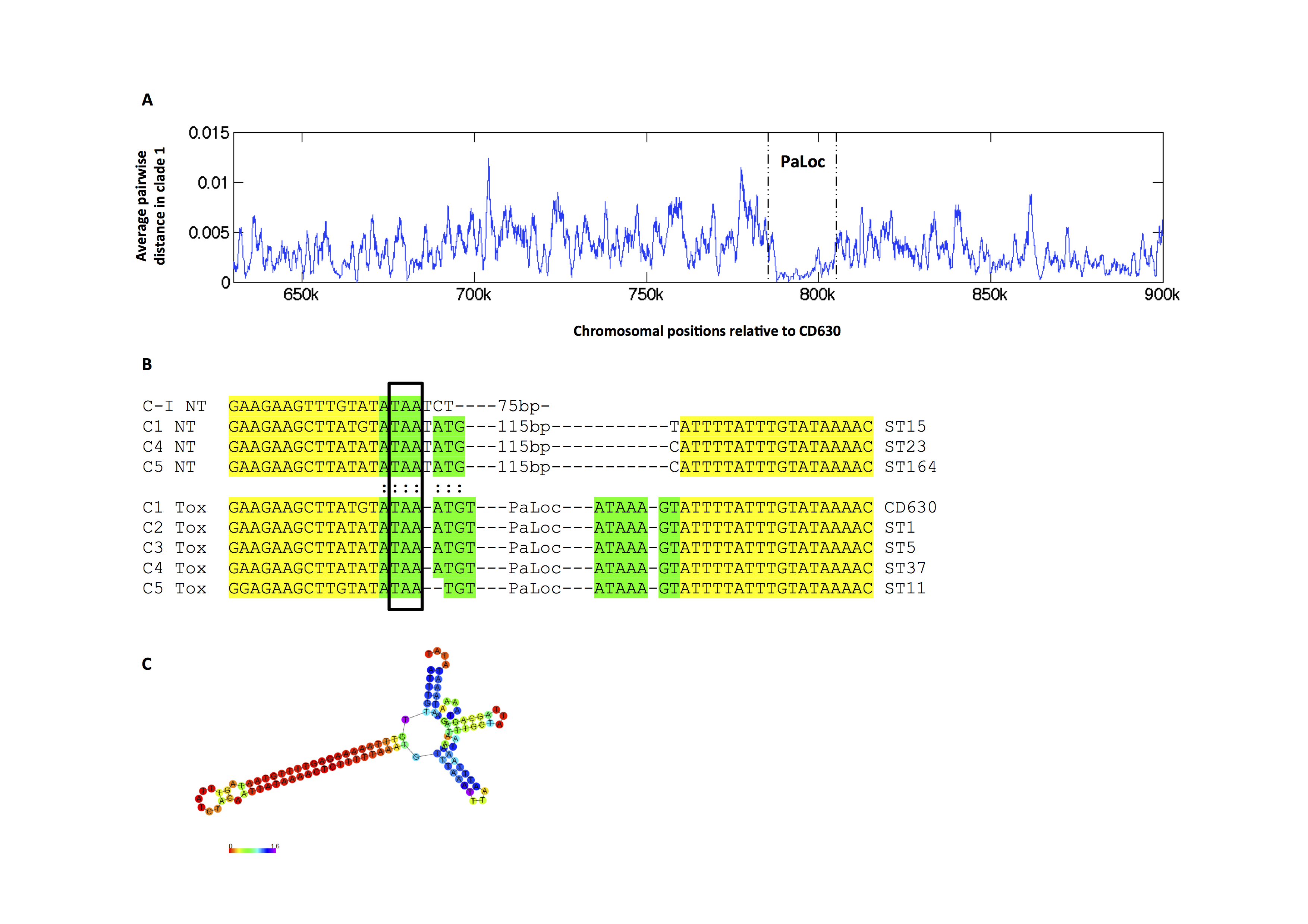

Supplement: Supplementary Data [file supp_evt204_Figure_S3_final.pptx.tif]

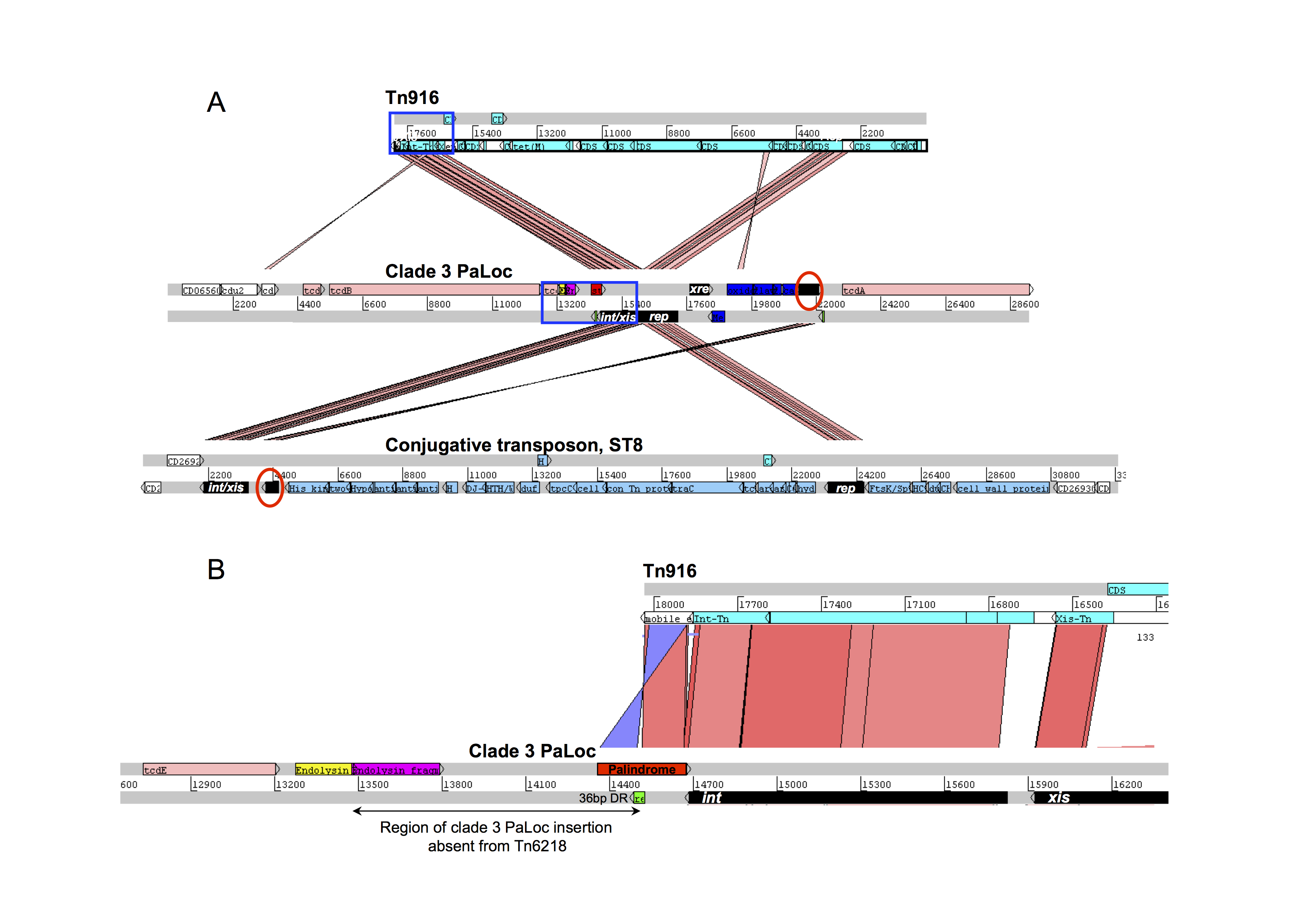

Supplement: Supplementary Data [file supp_evt204_Figure_S4_final.pptx.tif]

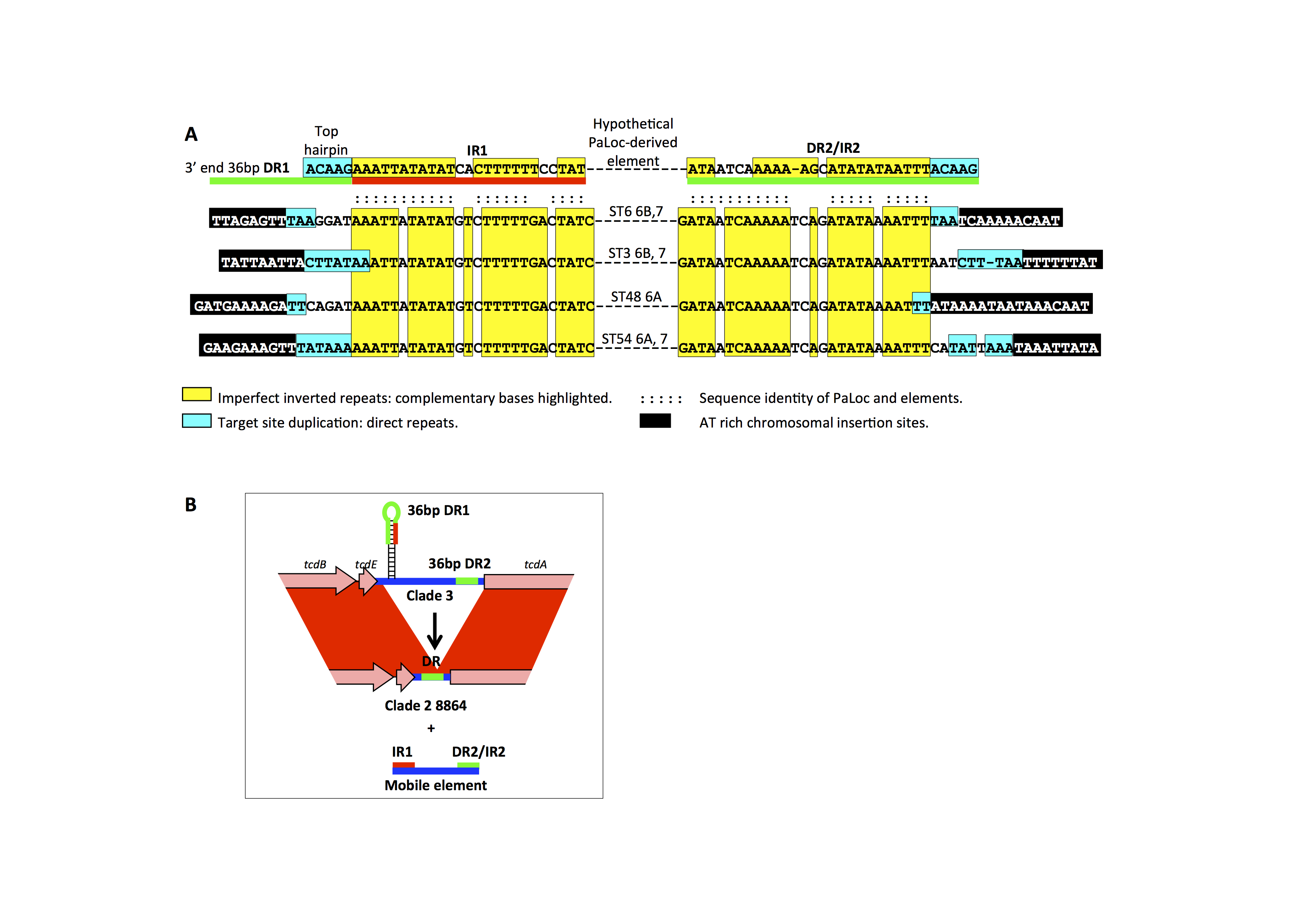

Supplement: Supplementary Data [file supp_evt204_Figure_S5_final.pptx.tif]

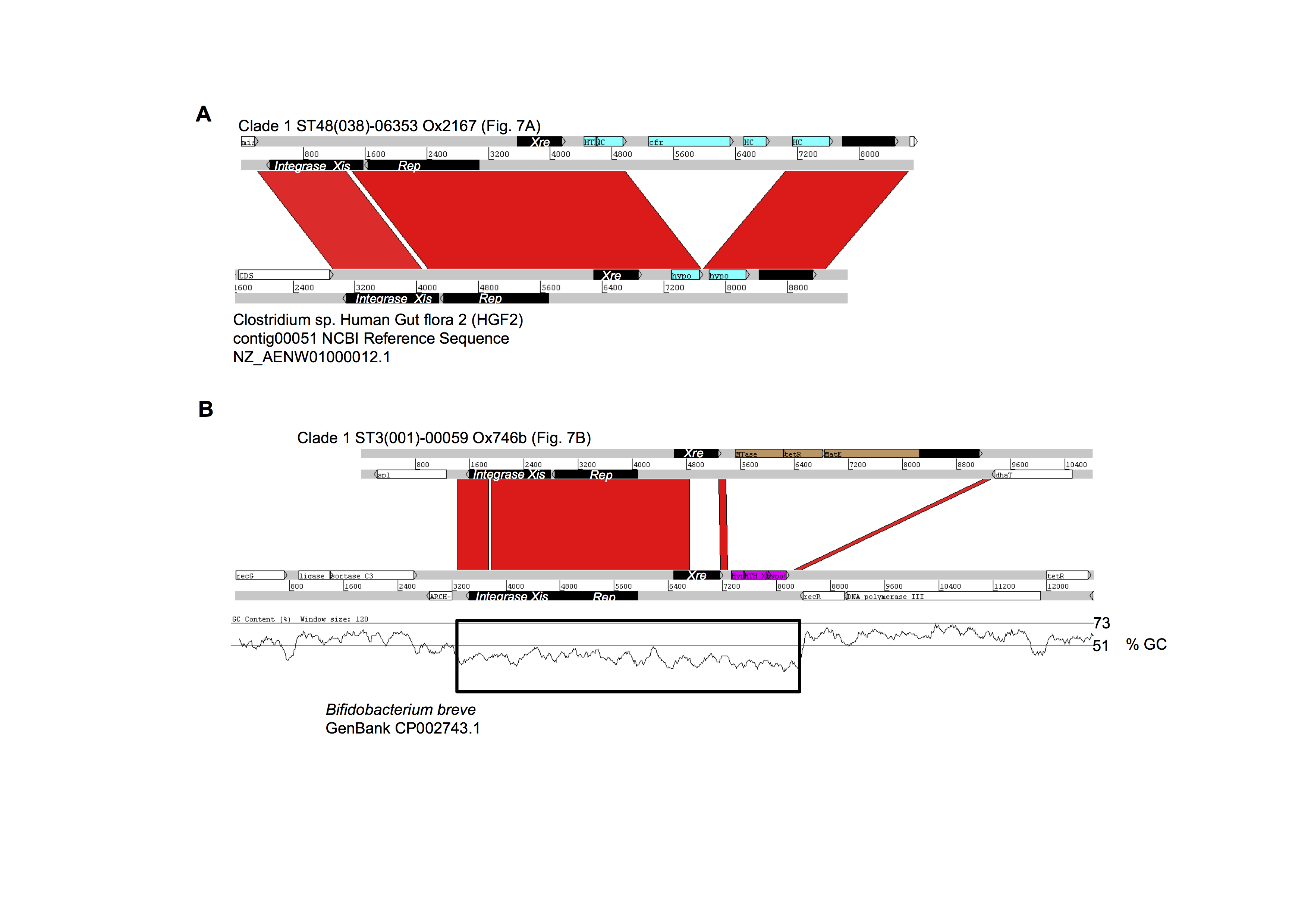

Supplement: Supplementary Data [file supp_evt204_Figure_S6_final.pptx.tif]
